# A64 VALIDATION OF THE MODIFIED MULTIPLIER OF SES-CD (MM-SES-CD) TO PREDICT ENDOSCOPIC REMISSION IN CROHN’S DISEASE: A POST HOC ANALYSIS OF THE SEAVUE TRIAL

**DOI:** 10.1093/jcag/gwae059.064

**Published:** 2025-02-10

**Authors:** S Anvari, D Ahuja, E Wong, P Dubai, J Marshall, V Jairath, W Reinisch, N Narula

**Affiliations:** McMaster University Faculty of Health Sciences, Hamilton, ON, Canada; Indira Gandhi National Open University, New Delhi, Delhi, India; McMaster University Faculty of Health Sciences, Hamilton, ON, Canada; Northwestern University, Evanston, IL; McMaster University Faculty of Health Sciences, Hamilton, ON, Canada; Western University, London, ON, Canada; Medizinische Universitat Wien Universitatsklinik fur Innere Medizin III, Wien, Wien, Austria; McMaster University Faculty of Health Sciences, Hamilton, ON, Canada

## Abstract

**Background:**

The modified multiplier of the SES-CD (MM-SES-CD) has been shown to have prognostic value for predicting endoscopic healing (EH) in patients with Crohn’s disease.

**Aims:**

The purpose of this analysis was to validate baseline categories of endoscopic disease severity using the MM-SES-CD and determine their prognostic value for predicting one-year EH.

**Methods:**

Participants in the SEAVUE trial (n = 386) were categorized based on baseline endoscopic disease severity using MM-SES-CD cut-offs into mild (≥22.5 to < 31), moderate (≥ 31 to < 45) and severe (≥45) disease. The primary outcome was achievement of endoscopic healing (EH) based on MM-SES-CD score (< 22.5) at one year. Secondary outcomes included achieving clinical remission based on patient-reported outcomes and normalization of fecal calprotectin in patients with raised baseline levels at one year.

**Results:**

MM-SES-CD < 22.5 at one year was achieved in 62.0% of patients with baseline mild endoscopic disease, 48.6% with moderate disease and 33.8% with severe disease (p <0.001). Similarly, a trend was observed for patient-reported outcome (PRO-2) clinical remission, which was achieved in 78.9% of patients with baseline mild endoscopic disease, 72.9% of those with moderate and 66.2% of those with severe disease (p=0.09). Likelihood of fecal calprotectin (FCP) normalization was significantly associated with baseline endoscopic disease severity (p=0.008).

**Conclusions:**

Baseline MM-SES-CD-based cutoffs for endoscopic disease severity demonstrate prognostic value for achieving one-year EH, PRO2 remission, and FCP normalization. These findings suggest that the MM-SES-CD can be used both to measure baseline endoscopic disease severity and predicts post-maintenance outcomes in patients with CD.

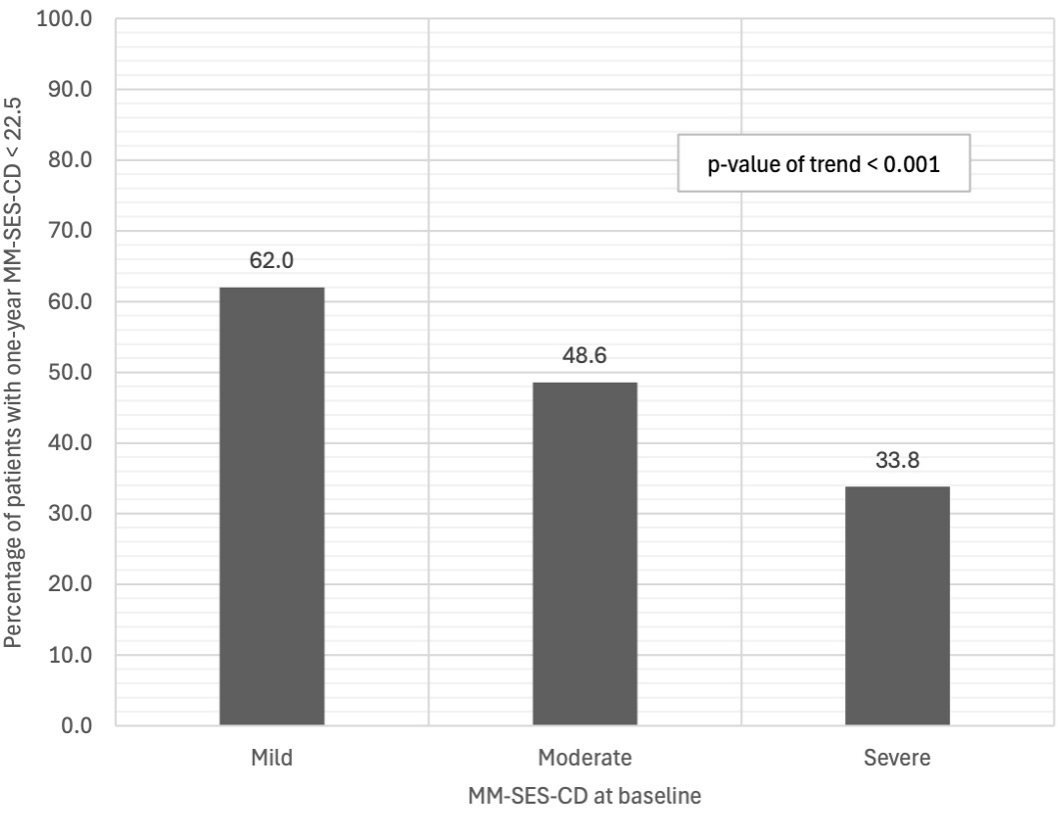

**Figure 1:** Percentage of patients in MM-SES-CD remission (<22.5) at week 52 stratified by baseline MM-SES-CD categories (Mild ≥ 22.5 to < 31, moderate ≥ 31 to < 45, severe ≥ 45)

**Funding Agencies:**

